# A Novel Long Noncoding RNA–LNC000133 Associated With Steroid‐Induced Osteonecrosis of the Femoral Head Promotes Osteoblast Differentiation Through Bone Marrow Mesenchymal Stem Cells‐Derived Exosomes Pathway: A Bioinformatics Validation and Detailed Mechanistic Study

**DOI:** 10.1111/jcmm.71135

**Published:** 2026-04-17

**Authors:** Chengbin Yang, Tixiong Xia, Xi Li, Tong Chen, Yingxing Xu

**Affiliations:** ^1^ Department of Orthopaedics First Affiliated Hospital of Kunming Medical University Kunming Yunnan China; ^2^ Research Center for Clinical Medicine First Affiliated Hospital of Kunming Medical University Kunming Yunnan China

**Keywords:** bone marrow‐derived mesenchymal stem cells, differential gene expression profiles, exosomes, LncRNA LNC000133, osteogenesis

## Abstract

Steroid‐induced osteonecrosis of the femoral head (SONFH) is a debilitating disease caused by glucocorticoid abuse, characterized by complex pathogenesis and unclear molecular mechanisms. Dysfunction of bone marrow mesenchymal stem cells (BMSCs) and their exosome‐mediated signalling is a key contributor to SONFH, although the precise mechanisms remain to be elucidated. In this study, the differential expression profiles of long noncoding RNAs (lncRNAs), microRNAs (miRNAs) and messenger RNAs (mRNAs) in exosomes derived from human BMSCs (hBMSCs) obtained from patients with SONFH compared to controls with femoral neck fractures were identified. Through next‐generation sequencing, a novel lncRNA, LNC000133, associated with SONFH was discovered. Using Gene Ontology (GO), Kyoto Encyclopedia of Genes and Genomes (KEGG) pathway analysis and competing endogenous RNA (ceRNA) network construction, the LNC000133/miR‐362‐5p/TGF‐β3/SMAD3/BMP2 signalling axis was established. The definitive expression, localization and full‐length sequence of LNC000133 in BMSCs were subsequently validated by Northern blot, quantitative real‐time polymerase chain reaction (qRT‐PCR), fluorescence in situ hybridization (FISH) and rapid amplification of cDNA ends (RACE). Most notably, mechanistic studies demonstrated that LNC000133‐modified BMSCs‐derived exosomes were efficiently taken up by osteoblasts, which promoted proliferation and osteogenic differentiation by targeting the miR‐362‐5p/TGF‐β3/SMAD3/BMP2 signalling pathway.

## 1 Introduction

Steroid‐induced osteonecrosis of the femoral head (SONFH) is a major subtype of non‐traumatic osteonecrosis associated with glucocorticoid abuse, characterized by increasing incidence, significant disability and frequent bilateral involvement [1, 2]. Its pathogenesis is complex and remains unclear, involving impaired bone remodelling, microcirculation disturbances and lipid metabolism disorders caused by the disruption of blood supply to the femoral head, posing a major challenge for hip preservation treatment in SONFH [3, 4].

Exosomes are nanoscale vesicles, typically 50 to 200 nm in diameter, released by various cell types [5, 6]. Recently, exosomes derived from bone marrow mesenchymal stem cells (BMSCs‐Exo) have gained increasing attention as key mediators of bone metabolism and tissue repair, due to their excellent biocompatibility and the capacity to deliver bioactive molecules, including long noncoding RNAs (lncRNAs), microRNAs (miRNAs), messenger RNAs (mRNAs) and proteins, to target cells [7]. Previous studies have confirmed the osteogenic potential of BMSCs‐Exo and identified several involved signalling pathways, such as Wnt/β‐catenin, BMP/Smad and PI3K/Akt [8, 9, 10]. However, the detailed molecular mechanisms remain largely unknown.

While our prior study revealed differential expression profiles of lncRNAs and mRNAs in BMSCs following exposure to high‐dose dexamethasone [11], it did not investigate exosomes associated with SONFH. Therefore, in this study, next‐generation sequencing (NGS) was employed to identify differentially expressed genes (DEGs) in exosomes derived from human BMSCs (hBMSCs‐Exos) obtained from patients with SONFH compared to control patients with femoral neck fracture. A novel lncRNA, LNC000133, linked to SONFH was identified, and its potential involvement in the miR‐362‐5p/TGF‐β3/SMAD3/BMP2 signalling pathway axis was predicted through Gene Ontology (GO), Kyoto Encyclopedia of Genes and Genomes (KEGG) pathway analysis and competing endogenous RNA (ceRNA) interaction network construction. Subsequently, the gene expression, cellular localization and full‐length sequence of LNC000133 in BMSCs were characterized by Northern blot, quantitative real‐time polymerase chain reaction (qRT‐PCR), fluorescence in situ hybridization (FISH) and rapid amplification of cDNA ends (RACE), respectively. Furthermore, mechanistic studies were conducted to investigate whether LNC000133‐modified hBMSCs‐Exos promote osteogenic differentiation by targeting the miR‐362‐5p/TGF‐β3/SMAD3/BMP2 signalling axis, while enhancing exosome uptake by osteoblasts.

## 2 Materials and Methods

### 2.1 Isolation and Culture of Human Bone Marrow Mesenchymal Stem Cells (hBMSCs)

This study was approved by the Ethics Committee of the First Affiliated Hospital of Kunming Medical University, and written informed consents were obtained from all patients. hBMSCs were isolated from femoral bone marrow aspiration fluid collected intraoperatively from patients with SONFH or femoral neck fractures undergoing total hip arthroplasty (THA). Cell isolation and purification were performed using density gradient centrifugation, as previously described [12]. When the cell confluence reached approximately 90%, cells were passaged at a 1:2 ratio. All experiments were conducted using cells at the third passage.

### 2.2 Phenotypes and Multilineage Differentiation of the hBMSCs

The surface markers of the BMSCs were analyzed using a flow cytometer (ACEA NovoCyte 2060R, ACEA Biosciences, USA). After trypsin digestion, cells were resuspended in phosphate‐buffered saline (PBS) containing 1% foetal bovine serum (FBS) and adjusted to a concentration of 1 × 10^6^ cells/mL. The cells were then incubated with anti‐CD34‐FITC, anti‐CD45‐FITC, anti‐CD73‐FITC and anti‐CD90‐FITC antibodies (BD Biosciences, USA) at 37°C in the dark for 30 min. Following incubation, the cells were washed with PBS, and flow cytometric analysis was performed. Unstained cells served as negative controls.

In addition, the osteogenic and adipogenic differentiation potential of the third‐passage hBMSCs was evaluated using differentiation media (Fuyuanbio, Shanghai, China), according to the established methods [11].

### 2.3 Isolation and Characterization of hBMSCs‐Exos

When hBMSCs reached 80%–90% confluence, they were washed with PBS and cultured in serum‐free medium (Yocon Biotech Co. Ltd., Beijing, Cat# NC0103). The cell culture supernatant was collected every 24 h and stored at 80°C. Exosomes were then isolated from the supernatant by ultracentrifugation [13].

Exosome morphology was analyzed using a transmission electron microscopy (TEM) analysis kit (E1610, Weihui Biotechnology, Beijing, China) following the previously reported methods [14], and observed and imaged using a TEM instrument (HITACHI, Japan, HT7800).

Particle size and concentration of the exosomes were measured by nanoparticle tracking analysis (NTA) using the ZetaView PMX 110 system (Particle Metrix, Meerbusch, Germany) according to established protocols [15].

Additionally, the exosomal marker proteins TSG101 and CD81 were detected by Western blot analysis.

### 2.4 RNA Sequencing

Total RNA was isolated from exosomes using the RNAiso Plus kit (Takara Bio Inc., Kusatsu, Japan) and reverse‐transcribed into complementary DNA (cDNA) as previously described [16]. Following standard protocols, ribosomal RNA (rRNA) was depleted, and the RNA was fragmented. First‐ and second‐strand cDNA synthesis was then performed, followed by end repair, 3′ adenylation, adaptor ligation and PCR enrichment to construct sequencing libraries. Library concentration were measured using a Qubit 2.0 Fluorometer, fragment size was assessed with an Agilent 2100 system and qRT‐PCR was used for accurate quantification of the effective library concentration. Qualified libraries were pooled based on concentration and desired sequencing output, and then subjected to Illumina sequencing. Differential expression analysis was conducted using the R package DESeq2, with DEGs defined as those with a fold change greater than 2 and a *P*‐value less than 0.05.

### 2.5 Bioinformatics Analysis

GO and KEGG pathway enrichment analyses of DEGs were performed using DAVID (version 6.8). GO analysis was conducted to identify the functions of these genes in terms of biological processes (BP), cellular components (CC) and molecular functions (MF), while KEGG analysis provided insights into the associated signalling pathways.

Furthermore, target miRNAs of LNC000133 and their binding sites were predicted using the miRDB website (https://mirdb.org) based on the initial sequences obtained from RNA sequencing. Subsequently, the ceRNA interaction network of LNC000133 was constructed using the ENCORI website (http://starbase.sysu.edu.cn/index.php) and visualized with Cytoscape version 3.7.2 software (NRNB, USA) [17].

### 2.6 Bioinformatics Validation of LNC000133

Northern blot was used to validate LNC000133 expression and determine its molecular size. Briefly, a total of 20 μg RNA was separated using denaturing polyacrylamide gel electrophoresis containing 7.5 M urea and 12% formaldehyde, and then transferred onto a Hybond‐*N* ^+^ nylon membrane (Amersham, Freiburg, Germany). The membrane was UV‐crosslinked for 2 min to immobilize the RNA, followed by hybridization with an antisense DNA probe targeting TPRG1‐AS1. After hybridization, the membrane was washed twice at 42°C in 2× Saline‐Sodium Citrate (SSC) buffer with 0.1% SDS (Invitrogen Life Technologies, Carlsbad, CA) for 20 min each wash. Signal detection was achieved by exposing the membrane to Kodak XAR‐5 film (Sigma‐Aldrich) [18]. A human U6 probe was used as a positive control in all assays. The sequences of all probes are provided in Table S1.

RACE was used to obtain the full‐length sequence of LNC000133. The 5′ RACE assay was conducted using the SMARTer RACE cDNA Amplification Kit (Takara Bio USA Inc., San Jose, CA, USA), following the manufacturer's protocols for reverse transcription and PCR amplification. Gene‐specific primers (GSPs) used for the reactions are listed in Table S2. For 3′ RACE, nested PCR was performed using an external primer (3′ GSP1) and an internal primer (3′ GSP2), with the amplification carried out according to the kit instructions [19]. All GSP sequences are provided in Table S2.

FISH was used to determine the subcellular localization of LNC000133 in hBMSCs, using the FISH kit (RiboBio, Guangzhou, China). Briefly, 5 × 10^4^ cells were seeded onto slides in 24‐well plates. After 24 h, when cells reached 60%–70% confluence, they were fixed with 4% paraformaldehyde and prehybridized at 37°C for 30 min. Hybridization with probes for LNC000133, U6 and 18S was performed overnight at 37°C in the dark. Subsequently, cells were washed three times at 42°C with 4× SSC (0.1% Tween‐20), 2× SSC and 1× SSC buffers, and then stained with DAPI for 10 min in the dark. After three PBS washes, fluorescence was detected by laser scanning confocal microscopy [20].

qRT‐PCR was used to assess the differential expression of LNC000133 in hBMSCs across different groups.

### 2.7 Cell Transfection

Recombinant lentiviruses for LNC000133 overexpression (up‐LNC000133), short‐hairpin RNA‐mediated knockdown (sh‐LNC000133) and their respective negative controls (Vector and sh‐Control) were constructed by OBiO Technology (Shanghai, China). miR‐362‐5p mimics and inhibitors were purchased from RiboBio (Guangzhou, China). hBMSCs were transfected with these reagents following the previously reported methods [16]. Transfection efficiency was evaluated by qRT‐PCR after 72 h. Subsequently, hBMSCs‐Exos were isolated as described earlier. Three samples were analyzed per group, and all experiments were performed in triplicate.

### 2.8 Cellular Uptake of Exosomes Derived From hBMSCs

Human immortalized osteoblasts were purchased from Cellverse Co. Ltd. (Shanghai, China) and seeded in 12‐well plates. When cells reached approximately 60% confluence, hBMSCs‐Exos were labelled with DiR dye (YEASEN, Shanghai, Cat. No. HB220509) following the manufacturer's instructions. Briefly, 100 μL of DiR working solution was added to 300 μg of exosome suspension and incubated at 37°C for 30 min with gentle vortexing. The mixture was then diluted with 10 mL of PBS and ultracentrifuged to remove excess dye [13]. The resulting DiR‐labelled hBMSCs‐Exos pellets were resuspended in 200 μL of PBS and co‐incubated with osteoblasts for 24 h. Uptake of hBMSCs‐Exos by osteoblasts was observed using a laser confocal microscope (Nikon, Japan, 200×, NIS‐Elements 5.11).

### 2.9 Crystal Violet Staining

Crystal violet staining was used to evaluate osteoblast proliferation. Cells were seeded in 96‐well plates and, upon reaching approximately 30% confluence, treated with hBMSCs‐Exos in each group. After incubation for 1 to 7 days, the cells were washed with PBS, fixed with 10% methanol for 15 min, and stained with 0.1% crystal violet for 15 min. Following three washes, 200 μL of 10% acetic acid was added and then incubated at 37°C for 20 min to dissolve the dye. Absorbance was measured at 595 nm using a microplate reader (Yongchuang, Shanghai, SM600).

### 2.10 Alkaline Phosphatase (ALP) Staining Assay

ALP staining was performed using a commercial kit (Solarbio, Beijing, China) to assess the osteogenic differentiation of osteoblasts. Briefly, cells were seeded in 24‐well plates and treated with hBMSCs‐Exos for 5 days. The cells were then fixed with 200 μL of 4% paraformaldehyde for 15 min, followed by incubation with the ALP working solution at 37°C in the dark for 20 min. Subsequently, nuclear fast red was applied for 5 min. Cells were observed under a light microscope, where blue staining indicated ALP activity [21]. PBS washes were performed three times before each step, and all groups were tested in triplicate.

### 2.11 ALP Activity Assay

ALP activity in hBMSCs and osteoblasts was measured using the ALP Activity Assay Kit (Nanjing Jiancheng Bioengineering Institute, Nanjing, China). As previously described [21], cells were seeded in 96‐well plates and treated with hBMSCs‐Exos for 5 days. After treatment, 500 μL of buffer and 500 μL of substrate solution were added to each well and incubated at 37°C for 15 min. Subsequently, 1.5 mL of chromogenic solution (alkaline solution containing 4‐aminoantipyrine and potassium ferricyanide) was added to develop colour. Absorbance was measured at 520 nm using a microplate reader, and ALP activity was calculated according to the manufacturer's instructions. PBS washes were performed three times at each step, and all groups were tested in triplicate.

### 2.12 Alizarin Red S (ARS) Staining Assay

ARS staining was performed to assess the osteogenic differentiation of osteoblasts. As previously described [11], cells were seeded in 24‐well plates and treated with hBMSCs‐Exos for 7 days. After treatment, cells were fixed with 200 μL of 4% paraformaldehyde for 20 min, followed by staining with 0.2% ARS solution for 15 min. After three washes with PBS, mineralized nodules were observed under an inverted phase‐contrast microscope. All experiments were performed and analyzed in triplicate.

### 2.13 qRT‐PCR

qRT‐PCR was performed according to previously described protocols [16]. All forward and reverse primers were provided by Ribobio Corporation (Guangzhou, China) and are listed in Table S3. Relative gene expression levels were calculated using the 2^−ΔΔCt^ method. GAPDH served as the internal control for LNC000133, TGF‐β3, SMAD3, BMP2, Runx‐2, BSP II and OPN3 mRNAs, while U6 was used to normalize miR‐362‐5p expression. Each group contained three samples, and all experiments were performed in triplicate.

### 2.14 Western Blotting Analysis

Total protein extraction and Western blot analysis of osteoblasts and hBMSCs‐Exos were performed as previously described [22]. Briefly, total protein was extracted from treated hBMSCs using pre‐chilled RIPA lysis buffer containing 1% protease inhibitor. Protein concentration was measured using a BCA assay. Samples were mixed with loading buffer (1:5) and heated at 95°C for 5 min. Equal amounts of protein (50 μg) were separated by 12.5% sodium dodecyl sulphate polyacrylamide gel electrophoresis (SDS‐PAGE) and transferred to polyvinylidene fluoride (PVDF) membranes. Membranes were blocked with 5% non‐fat milk in TBST for 2 h, incubated with primary antibodies overnight at 4°C, and then with horseradish peroxidase‐conjugated secondary antibodies at 37°C for 1 h. Protein bands were visualized using enhanced chemiluminescence PLUS (ECL‐PLUS) reagent and imaged with a chemiluminescence detection system. Band intensities were quantified using ImageJ software and normalized to GAPDH. Primary antibodies against TSG101 and CD81 were purchased from Proteintech (Wuhan, China), while antibodies against OPN3, BSP II and Runx‐2 were obtained from Affinity Biosciences (Cincinnati, USA). The GAPDH primary antibody and all secondary antibodies were purchased from ABclonal (Wuhan, China).

### 2.15 Dual‐Luciferase Reporter Assay System

All GV272‐LNC000133 plasmids (including GV272‐LNC000133‐WT and GV272‐LNC000133‐MUT) and the Renilla luciferase reporter plasmid were provided by RioBio (Guangzhou, China). Third‐passage cells were seeded into 96‐well plates at a density of 2 × 10^4^ cells per well and transfected with GV272‐LNC000133 plasmids, Renilla luciferase plasmid, miR‐362‐5p mimics, inhibitors, or miR‐negative control (NC) using Lipofectamine 2000. After 48 h, luciferase activity was measured using the Dual‐Luciferase Reporter Assay Kit (Yeasen Biotech, Shanghai) according to the manufacturer's instructions.

### 2.16 AGO2‐RIP Assay

The RIP assay was performed using a RIP kit (BersinBio, Guangzhou, China) according to the manufacturer's instructions. Briefly, 4 × 10^7^ cells were lysed in polysome lysis buffer containing protease and RNase inhibitors, and were incubated with RIP buffer containing magnetic beads conjugated with anti‐AGO2 antibodies (Cell Signalling Technology, Danvers, USA) and immunoglobulin G (IgG) antibodies.

(BersinBio, Guangzhou, China) for 16 h at 4°C. To verify immunoprecipitation efficiency, a portion of the immunoprecipitated complexes was analyzed by western blot for Argonaute 2 (AGO2) enrichment. Then, proteinase K was used to digest the unbound proteins in the remaining immunoprecipitated complexes, and co‐immunoprecipitated RNA was isolated. Subsequently, the expression of LNC000133, miR‐362‐5p, GAPDH and U6 in each group was detected by qRT‐PCR.

### 2.17 RNA Pull‐Down Assay

The RNA pull‐down assay was performed using an RNA pull‐down kit (BersinBio, Guangzhou, China) according to the manufacturer's instructions. Briefly, biotin‐labelled miR‐362‐5p probe (5′‐AAUCCUUGGAACCUAGGUGUGAGU‐3′, 3′‐biotin) and the corresponding negative control probe were synthesized by GENCEFE (China). Approximately 4 × 10^7^ cells were lysed in lysis buffer containing protease and RNase inhibitors. The cell lysates were then incubated with the biotin‐labelled probes at 4°C for 4 h, followed by incubation with streptavidin‐coated magnetic beads for an additional 2 h at 4°C to capture the RNA–RNA complexes. To verify pull‐down efficiency, a portion of the complexes was analyzed by western blot for AGO2 enrichment. The remaining complexes were purified, and LNC000133 enrichment was assessed by qRT‐PCR.

### 2.18 Statistical Analysis

Data were analyzed using SPSS 19.0 software (IBM, USA). One‐way analysis of variance (ANOVA) was applied for comparisons among multiple groups, while independent‐sample *t*‐tests were used for comparisons between two groups. Quantitative data were expressed as mean ± standard deviation (SD). Levene's test assessed the homogeneity of variances; if variances were unequal, Tamhane's T2 post hoc test was used. A *p*‐value < 0.05 was considered statistically significant. Graphs were generated using GraphPad Prism 9 software (GraphPad, USA).

## 3 Results

### 3.1 Identification of the hBMSCs

**FIGURE 1:**
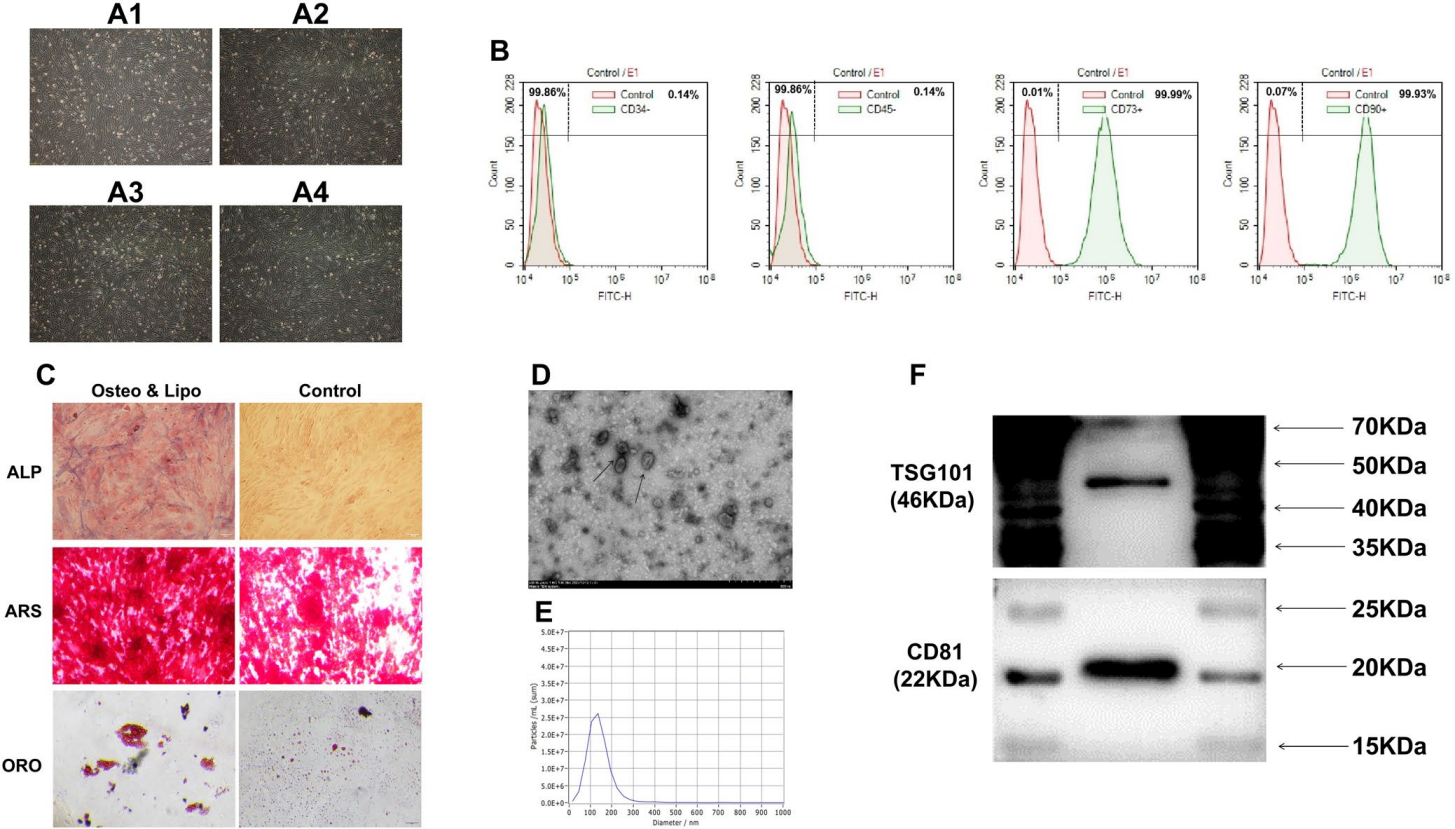
Characterization of the hBMSCs and hBMSCs‐Exos. (A) Morphology of hBMSCs at passages 3–4 (Scale bar: 200 μm); A1—A4, passage1—passage4; (B) Phenotypic analysis of hBMSCs by flow cytometry; (C) ALP staining, ARS staining and Oil Red O staining of hBMSCs (scale bar: 100 μm); (D) TEM analysis of exosomes; (E) Particle size‐concentration distribution of exosomes. The X‐axis (size/nm) indicates particle diameter; the Y‐axis (particles/mL) shows particle concentration. The peak value represents the most abundant particle size, while the peak width reflects size distribution uniformity; (F) WB analysis of exosome‐specific marker proteins. ALP, alkaline phosphatase; ARS, Alizarin Red S; hBMSCs‐Exos, exosomes derived from human BMSCs; hBMSCs, human bone marrow mesenchymal stem cells; ORO, Oil Red O; NTA, nanoparticle tracking analysis; TEM, transmission electron microscopy; WB, western blot.

The isolated cells at the third to fourth passages exhibited an elongated, spindle‐shaped morphology with clear boundaries, strong refractivity and a radial or whirlpool‐like arrangement (Figure 1A). Flow cytometry analysis demonstrated that the isolated cells were positive for the bone marrow‐derived stem cell surface markers CD73 (99.99%) and CD90 (99.93%), and negative for the haematopoietic‐specific surface markers CD34 (99.86%) and CD45 (99.86%) (Figure 1B). After adipogenic induction, both the number and size of lipid droplets increased significantly (Figure 1C). Following osteogenic induction, mineralized nodule formation and increased ALP activity were observed (Figure 1C).

### 3.2 Identification of hBMSCs‐Exos

TEM analysis revealed that the exosomes purified from the hBMSCs culture supernatants exhibited a typical saucer‐shaped morphology (Figure 1D). NTA showed that the particle size was primarily distributed between 50 to 200 nm, with a peak size of 138.3 nm, a median size of 114.7 nm and a concentration of 6.1 × 10^11^ particles/mL, consistent with the characteristic size of exosomes (Figure 1E). Western blot analysis further confirmed the expression of the exosome‐specific markers CD81 and TSG101 (Figure 1F).

### 3.3 Differential Expression Profiles of lncRNAs in hBMSCs‐Exos From SONFH Patients and Bioinformatics Analysis

A total of 319 differentially expressed lncRNAs, including 88 upregulated and 231 downregulated transcripts, were identified in hBMSCs‐Exos from patients with SONFH using NGS (Figure S1A,B). Among these, intronic lncRNAs were the most prevalent (70.8%), followed by long intergenic non‐coding RNAs (lincRNAs, 21%) and antisense lncRNAs (8.2%) (Figure S1G). Notably, novel lncRNAs constituted the majority of the differentially expressed transcripts, with 70% of the upregulated and 27% of the downregulated lncRNAs being unannotated while only a small portion were previously annotated (Figure S1H).

GO enrichment analysis showed that these differentially expressed lncRNAs were significantly involved in BPs such as cell cycle regulation, intracellular transport, post‐transcriptional gene regulation, DNA replication, gene expression, chromatin remodelling and both protein and nucleic acid metabolism (*p* < 0.05). In the MF category, the lncRNAs were enriched in protein binding, RNA binding, nucleosome binding, protein‐DNA complex binding, enzyme activity, ATP/GTP binding, ion binding and transmembrane transport (*p* < 0.05). Regarding cellular components (CC), enrichment was observed in the nucleus, nucleoplasm, chromatin, nucleosomes, protein–DNA complexes, membrane‐bound organelles, cytoplasm and protein complexes (*p* < 0.05) (Figure S1C,D).

KEGG pathway analysis indicated that these lncRNAs were associated with signalling pathways related to cell proliferation and differentiation, including PI3K‐Akt, Hippo, insulin, HIF‐1 and apoptosis pathways [23, 24]. In addition, pathways such as oxidative phosphorylation, glycolysis/gluconeogenesis and the pentose phosphate pathway were potentially linked to SONFH pathogenesis (Figure S1E,F).

### 3.4 Differential Expression Profiles of miRNAs in hBMSCs‐Exos From SONFH Patients and Bioinformatics Analysis

A total of nine differentially expressed miRNAs, including four upregulated and five downregulated, were identified in hBMSCs‐Exos from patients with SONFH using NGS (Figure S2A,B).

GO enrichment analysis revealed that the target mRNAs of these miRNAs were significantly involved in BPs related to cell proliferation and differentiation, including cellular metabolism, macromolecule metabolism, organic substance metabolism, primary metabolic processes, mitochondrial development and extracellular matrix organization. In the MF category, enriched terms included catalytic activity, small molecule binding, ATP binding, nucleotide binding, ion transport and transmembrane transporter activity. Regarding CCs, the targets were primarily localized to the cytoplasm, nucleus, organelles, membrane‐bound organelles, Golgi apparatus, plasma membrane and calcium channel complexes (Figure S2C).

KEGG pathway analysis further indicated significant enrichment in signalling pathways associated with cell proliferation and differentiation, including the mitogen‐activated protein kinase (MAPK) pathway, mechanistic target of rapamycin pathway and the neurotrophin signalling pathway [25, 26] (Figure S2D).

### 3.5 Differential Expression Profiles of mRNAs in hBMSCs‐Exos From SONFH Patients and Bioinformatics Analysis

A total of 394 differentially expressed mRNAs, including 66 upregulated and 328 downregulated transcripts, were identified in hBMSCs‐Exos from patients with SONFH using NGS (Figure S3A,B).

GO enrichment analysis revealed that these mRNAs were significantly enriched in BPs related to cell proliferation, differentiation and exosome uptake, such as cellular metabolism, cellular component organization, organelle organization, macromolecular and protein complex assembly and precursor metabolite generation (*p* < 0.05). In the MF category, enriched terms included RNA binding, protein binding and GTPase activity. Regarding CCs, the DEGs were primarily localized in the cytoplasm, membrane‐bound organelles, extracellular vesicles (including exosomes), vesicles and intracellular membranes (*p* < 0.05) (Figure S3C).

KEGG pathway analysis indicated significant enrichment in key signalling pathways related to cell proliferation and differentiation, including the MAPK signalling pathway, oxidative phosphorylation, biosynthesis of amino acids, ribosome biogenesis in eukaryotes and protein processing in the endoplasmic reticulum [27, 28]. Additionally, ubiquitin‐mediated proteolysis and adherens junction pathways may be involved in exosome uptake [29] (Figure S3D).

### 3.6 lncRNA‐miRNA‐mRNA Co‐Expression Network Analysis

**FIGURE 2:**
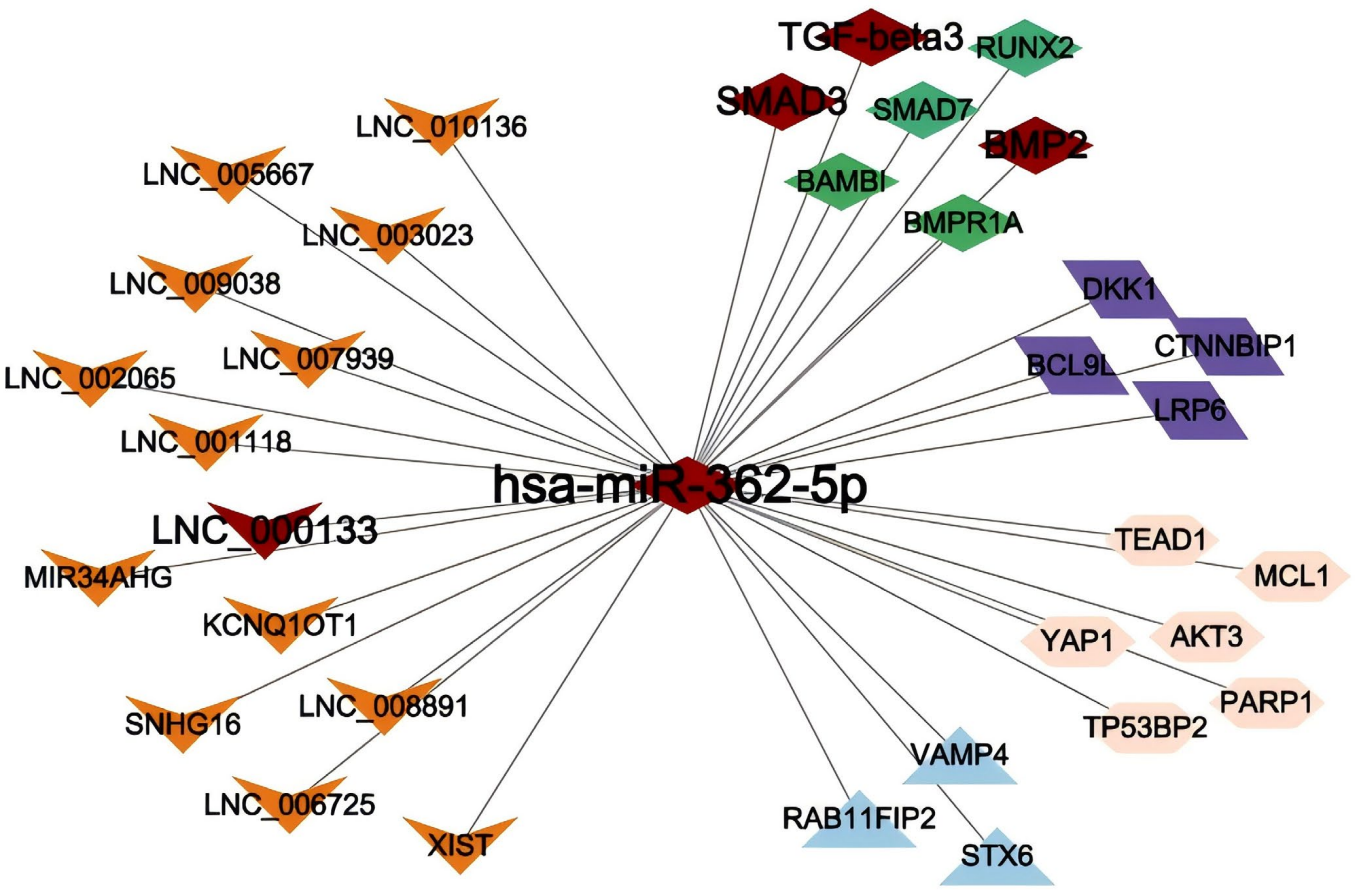
CeRNA regulatory network centred on hsa‐miR‐362‐5p. ceRNA, competing endogenous RNA; Orange V‐shaped nodes represent lncRNAs; the central red hexagon represents hsa‐miR‐362‐5p; mRNAs are represented by rhombus and triangle shapes in different colours. Green diamonds indicate genes in the TGF‐β signalling pathway; purple parallelograms indicate Wnt signalling pathway genes; blue triangles represent membrane trafficking‐related genes; and pink diamonds indicate genes involved in apoptosis or transcriptional regulation; All red shapes represent the signalling pathway axis investigated in this study. Edges denote predicted or validated interactions.

Preliminary sequencing results showed that LNC000133 was significantly downregulated in hBMSCs‐derived exosomes from SONFH patients, while miR‐362‐5p was upregulated. Concurrently, downstream osteogenic pathway‐related genes (TGF‐β3, SMAD3 and BMP2) were all downregulated, consistent with a typical ceRNA regulatory pattern. Based on structural complementarity, co‐expression and co‐localization analyses, it was predicted that LNC000133 may act as a molecular sponge for miR‐362‐5p, thereby relieving its inhibitory effect on these downstream target genes. Accordingly, a LNC000133/miR‐362‐5p/TGF‐β3/SMAD3/BMP2 ceRNA regulatory network was constructed (Figure 2), highlighting the potential role of this pathway in impaired osteogenesis in SONFH.

### 3.7 Bioinformatics Validation of LNC000133

**FIGURE 3:**
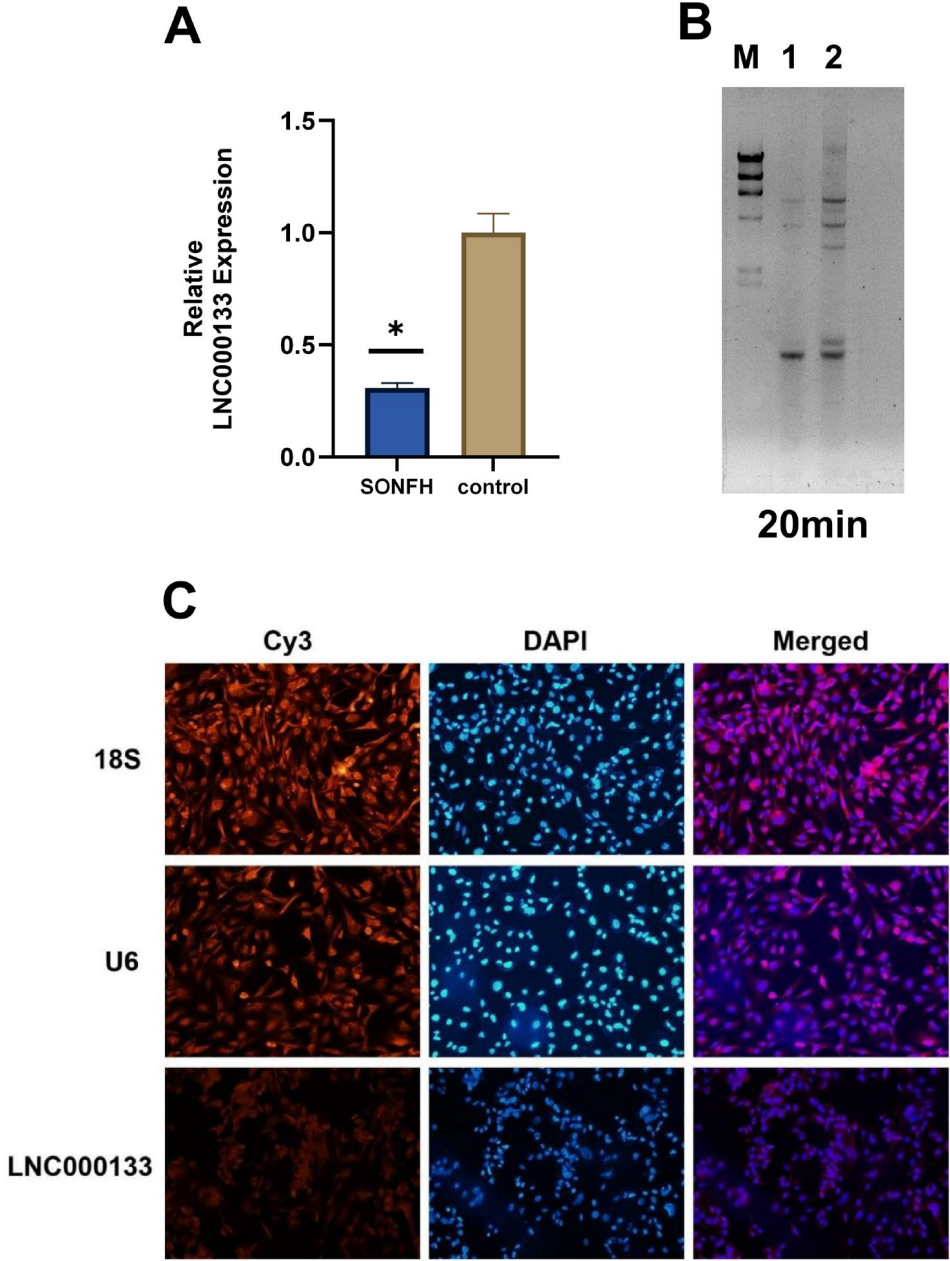
Bioinformatics validation of LNC000133. (A) qRT‐PCR analysis showing the differential expression of LNC000133 in hBMSCs from patients with SONFH and femoral neck fractures; (B) Northern blot analysis confirming the precise expression of LNC000133 in hBMSCs. M: DNA molecular weight marker II (564, 2027, 2322, 4361 bp); Lane 1: Cells; Lane 2: Cells; (C) FISH showing the localization of LNC000133 in hBMSCs. FISH: Fluorescence In Situ Hybridization; RACE, rapid amplification of cDNA ends; WB, Western Blot; *p < 0.05 compared with the control group.

qRT‐PCR analysis revealed that the expression level of LNC000133 in hBMSCs from SONFH patients was significantly lower than that in patients with femoral neck fractures, consistent with the sequencing results (Figure 3A). Northern blot analysis further confirmed the presence of LNC000133 in hBMSCs (Figure 3B). In addition, the full‐length sequence of LNC000133 was successfully obtained using RACE, and the assembled sequence is shown in Figure S4. Furthermore, FISH analysis demonstrated that, unlike U6 (primarily localized in the nucleus) and 18S (primarily localized in the cytoplasm), LNC000133 was distributed in both the cytoplasm and nucleus of most hBMSCs (Figure 3C).

### 3.8 LNC000133 Promoted the Uptake of hBMSCs‐Exos by Osteoblasts

**FIGURE 4:**
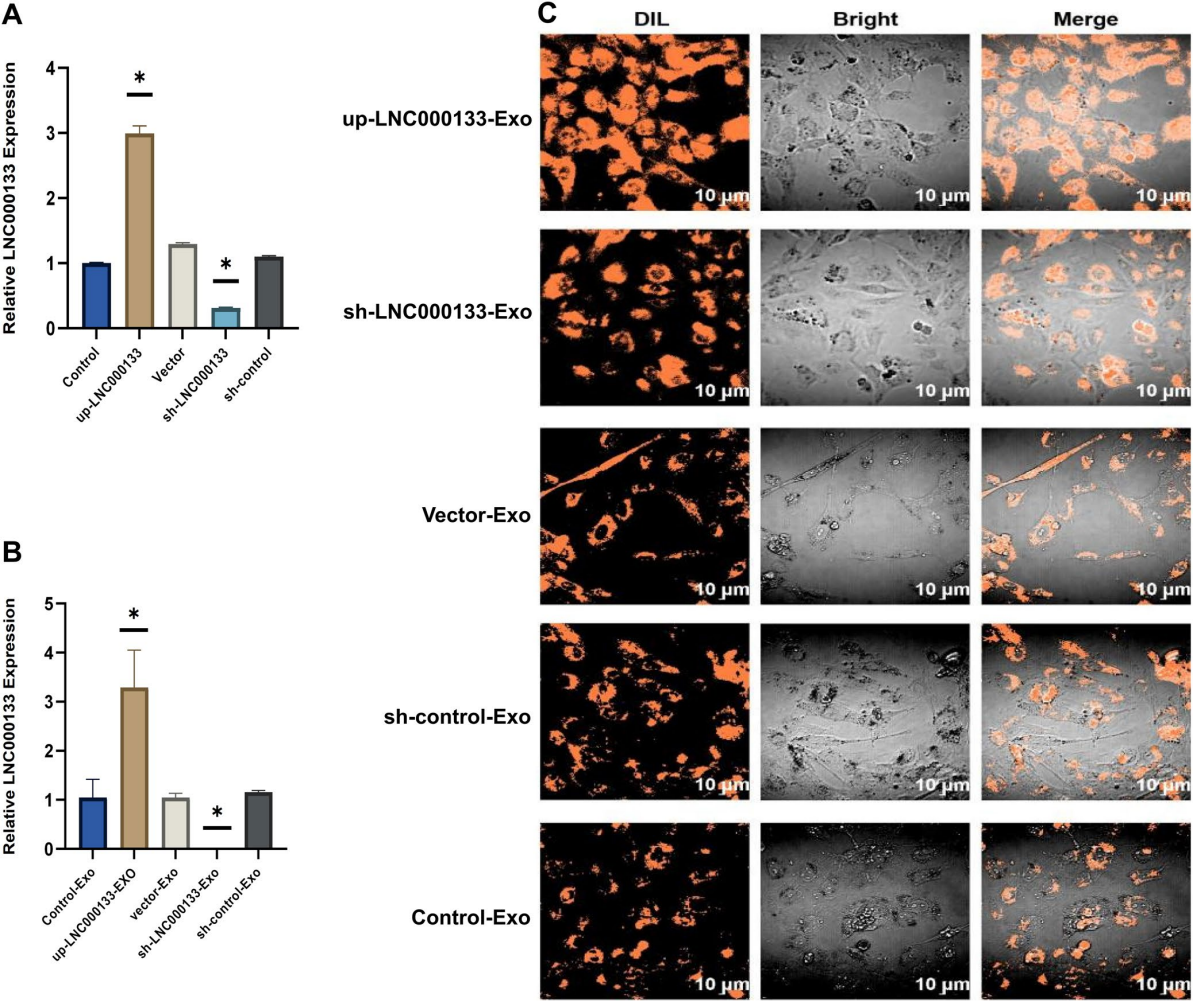
Cellular uptake of hBMSCs‐Exos. (A, B) qRT‐PCR analysis of LNC000133 expression in hBMSCs and hBMSCs‐Exos after lentiviral transduction; (C) Uptake of hBMSCs‐Exos by osteoblasts. Scale bar: 10 μm. *p < 0.05 compared with the control group.

Following infection with up‐LNC000133 or sh‐LNC000133 lentivirus, over 90% of hBMSCs exhibited green fluorescence, indicating high transfection efficiency (Figure S5). qRT‐PCR analysis confirmed that up‐LNC000133 lentivirus significantly increased LNC000133 expression in both hBMSCs and hBMSCs‐Exos, whereas sh‐LNC000133 lentivirus reduced its expression compared to controls (*p* < 0.05) (Figure 4A,B), demonstrating successful transfection.

Moreover, laser confocal microscopy revealed that osteoblasts in the up‐LNC000133 group internalized more DiR‐labelled hBMSCs‐Exos, while fewer DiR‐labelled exosomes were observed in the sh‐LNC000133 group compared with controls (Figure 4C). These results indicated that LNC000133 markedly enhanced the uptake of hBMSCs‐Exos by osteoblasts.

### 3.9 LNC000133‐Modified hBMSCs‐Exos Promoted the Proliferation and Osteogenic Differentiation of Osteoblasts

**FIGURE 5:**
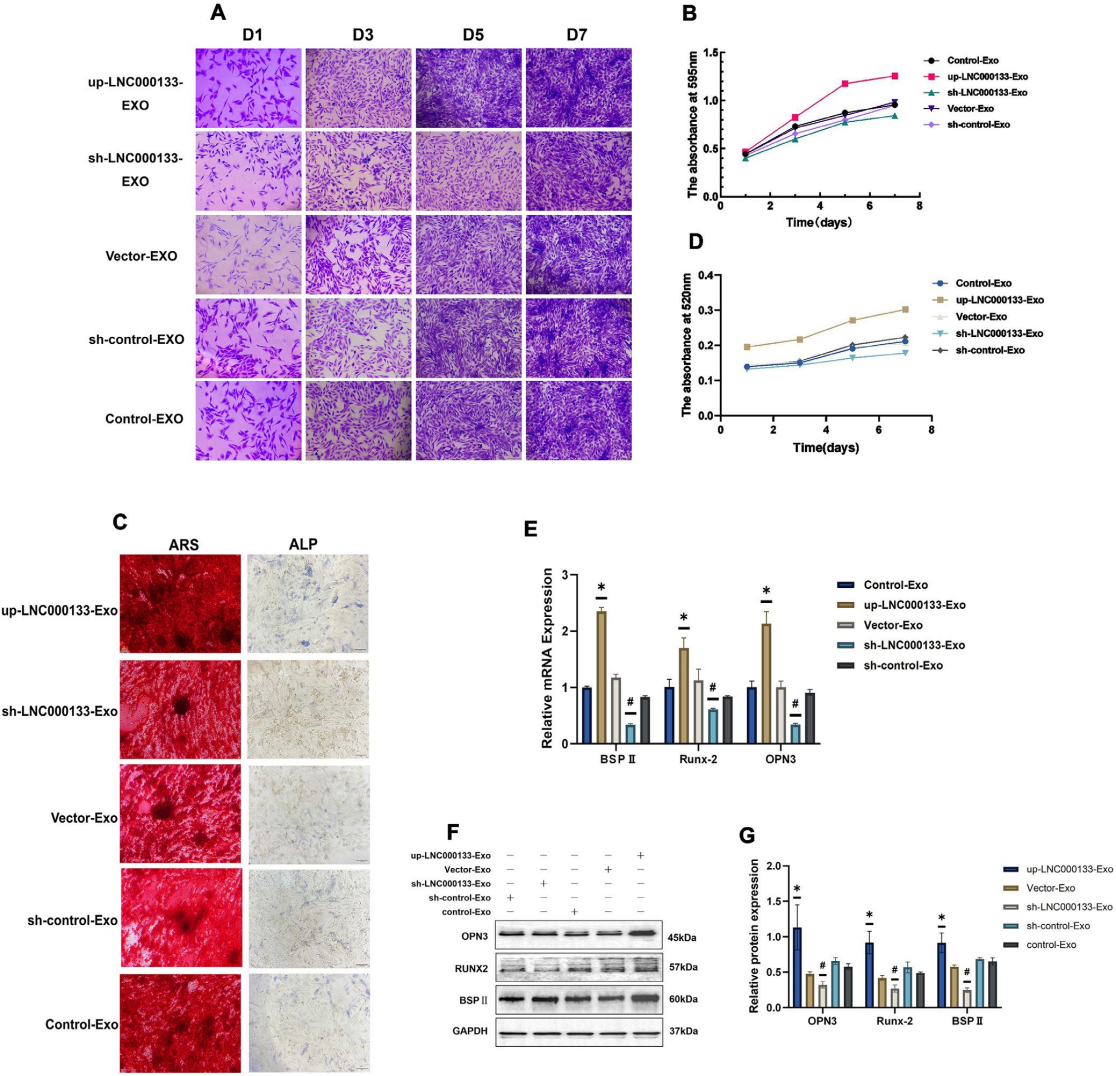
Functional validation of LNC000133. (A) Crystal violet staining; scale bar: 200 μm; (B) Proliferation viability of osteoblasts co‐incubated with hBMSCs‐Exos from day 1 to day 7, determined by crystal violet assay; (C) ARS staining and ALP staining of osteoblasts; scale bar: 100 μm; (D) ALP activity of osteoblasts from day 1 to day 7; (E) Expression levels of osteogenesis‐related mRNAs; (F) Representative Western blot bands of osteogenesis‐related proteins; (G) Quantification of osteogenesis‐related protein expression. *p < 0.05 compared with the vector‐Exo group; ^#^ *p* < 0.05 compared with the sh‐control‐Exo group. ALP, alkaline phosphatase; ARS, Alizarin Red S; BSPII, Bone sialoprotein II; OPN3, Opsin 3; Runx‐2, Runt‐related transcription factor 2.

Crystal violet staining analysis showed obvious cell growth in the up‐LNC000133‐Exo group, whereas delayed cell growth was observed in the sh‐LNC000133‐Exo group compared with the control groups (*p* < 0.05) (Figure 5A,B). These results demonstrated that LNC000133‐modified hBMSCs‐Exo significantly promoted osteoblast proliferation.

Additionally, ALP staining and activity assays revealed significantly higher ALP activity in the up‐LNC000133‐Exo group compared with the control group, while the sh‐LNC000133‐Exo group showed a trend toward inhibited ALP activity (*p* < 0.05) (Figure 5C,D). These findings indicated that LNC000133‐modified hBMSCs‐Exo markedly promoted the osteogenic differentiation of osteoblasts.

Alizarin red staining showed a greater number of mineralized nodules in the up‐LNC000133‐Exo group compared with controls, whereas the sh‐LNC000133‐Exo group exhibited impaired mineral nodule formation (*p* < 0.05) (Figure 5C). These results suggest that LNC000133‐modified hBMSCs‐Exo significantly enhance osteogenic differentiation.

Moreover, qRT‐PCR and Western blot analyses demonstrated that the expression levels of osteogenesis‐related genes and proteins, including Runx‐2, BSP II and OPN3, were markedly upregulated in the up‐LNC000133‐Exo group and significantly downregulated in the sh‐LNC000133‐Exo group compared with controls (*p* < 0.05) (Figure 5E–G).

Collectively, these results indicated that LNC000133‐modified hBMSCs‐derived exosomes significantly promoted osteoblast proliferation and osteogenic differentiation.

### 3.10 LNC000133‐Modified hBMSCs‐Exos Promoted the Proliferation and Osteogenic Differentiation of Osteoblasts by Activating the TGF‐β3/SMAD3/BMP2 Signalling Pathway

Based on previous bioinformatics analyses, LNC000133 may regulate key genes in the TGF‐β signalling pathway, such as TGF‐β3, SMAD3 and BMP2, by acting as a molecular sponge for miR‐362‐5p. KEGG pathway enrichment of miR‐362‐5p target genes further revealed significant enrichment of the TGF‐β signalling pathway, suggesting its potential involvement in the pro‐osteogenic effects of LNC000133. Therefore, the TGF‐β pathway was selected as a key target for subsequent functional validation to elucidate the mechanism of action of LNC000133‐modified hBMSCs‐Exo.

**FIGURE 6:**
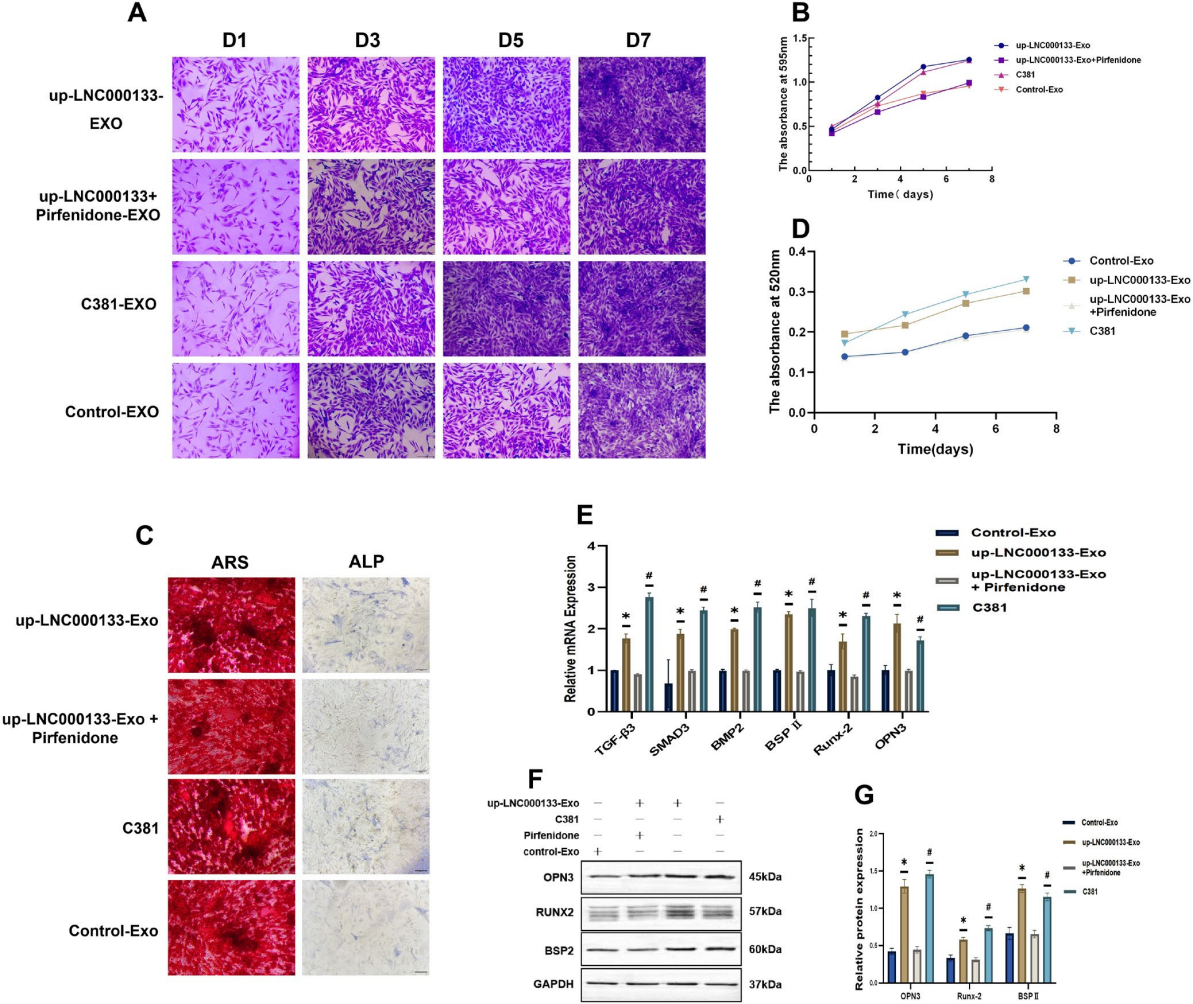
Results of TGF‐β3 activation and inhibition experiments. (A) Crystal violet staining; scale bar: 200 μm; (B) Proliferation viability of osteoblasts co‐incubated with hBMSCs‐Exos from day 1 to day 7, determined by crystal violet assay; (C) ARS staining and ALP staining of osteoblasts; scale bar: 100 μm; (D) ALP activity of osteoblasts from day 1 to day 7; (E) Expression levels of related mRNAs; (F) Representative Western blot bands of osteogenesis‐related proteins; (G) Quantification of osteogenesis‐related protein expression. *p < 0.05 compared with the up‐LNC000133‐Exo + Pirfenidone group; ^#^ *p* < 0.05 compared with the control‐Exo group. ALP, alkaline phosphatase; ARS, Alizarin Red S.

Crystal violet staining showed that cell proliferation in the C381 (an agonist for TGF‐β signalling pathway) group was comparable to that in the up‐LNC000133‐Exo group, whereas proliferation was significantly reduced in the up‐LNC000133‐Exo + Pirfenidone (an inhibitor for TGF‐β signalling pathway) group (*p* < 0.05) (Figure 6A,B), indicating that the TGF‐β signalling inhibitor Pirfenidone attenuates the pro‐proliferative effect of LNC000133‐modified hBMSCs‐Exo on osteoblasts.

ALP staining and activity assays demonstrated that the ALP activity levels in the C381 group were comparable to that in the up‐LNC000133‐Exo group, whereas the ALP activity was significantly reduced in the up‐LNC000133‐Exo + Pirfenidone group (*p* < 0.05) (Figure 6C,D), suggesting Pirfenidone significantly suppresses the pro‐osteogenic effects of LNC000133‐modified hBMSCs‐Exo on osteoblasts.

Alizarin red staining further revealed reduced mineralized nodule formation in the Pirfenidone‐treated group compared to the C381 and up‐LNC000133‐Exo groups (*p* < 0.05) (Figure 6C), confirming the involvement of the TGF‐β pathway in osteogenesis.

qRT‐PCR and Western blot analyses showed that the expression of TGF‐β3, SMAD3, BMP2, Runx‐2, BSP II and OPN3 was significantly upregulated in both the up‐LNC000133‐Exo and C381 groups, but was suppressed by Pirfenidone treatment, with levels returning to baseline (*p* < 0.05) (Figure 6E–G).

These results indicated that LNC000133‐modified hBMSCs‐Exos promoted the proliferation and osteogenic differentiation of osteoblasts by activating the TGF‐β3/SMAD3/BMP2 signalling pathway.

### 3.11 LNC000133 Targeted miR‐362‐5p as a ceRNA

FISH analysis showed that LNC000133 is localized in both the nucleus and cytoplasm of hBMSCs, suggesting a role in post‐transcriptional regulation. Considering that many lncRNAs act as ceRNAs, we investigated the potential interaction between LNC000133 and miR‐362‐5p.

**FIGURE 7:**
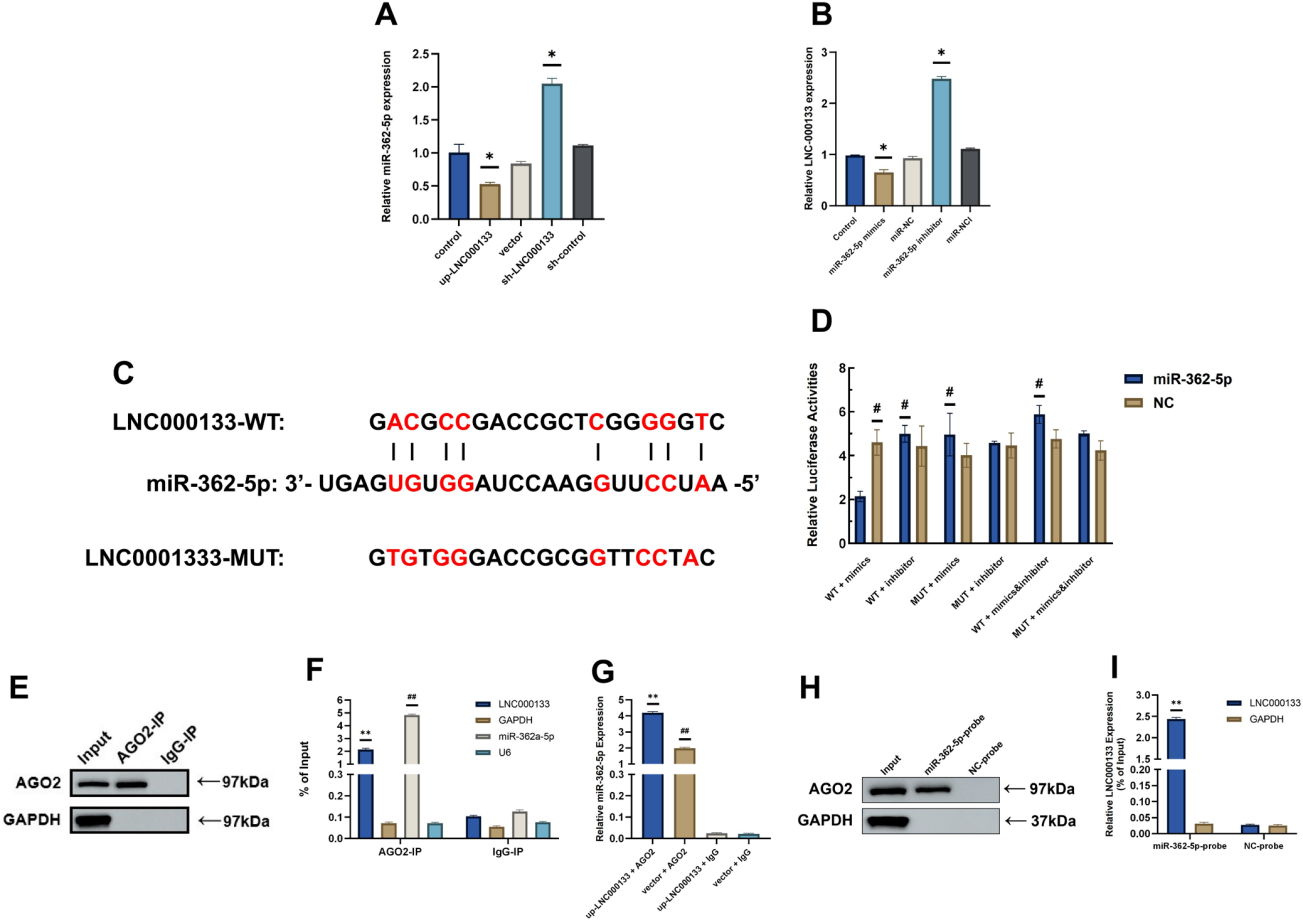
LNC000133 targeted miR‐362‐5p as a ceRNA. (A) qRT‐PCR analysis of the regulation of miR‐362‐5p expression by LNC000133; (B) qRT‐PCR analysis of the regulatory effect of miR‐362‐5p on LNC000133 expression; (C) Construction of WT and MUT LNC000133 sequences lacking the miR‐362‐5p binding site. (D) Relative luciferase activity after co‐transfection of miR‐362‐5p mimics with WT or MUT LNC000133 reporter constructs; (E) Western blot analysis of AGO2 protein to verify successful immunoprecipitation in the RIP assay; (F) qRT‐PCR showing enrichment efficiency of LNC000133 and miR‐362‐5p based on AGO2‐RIP; (G) qRT‐PCR showing enrichment efficiency of miR‐362‐5p after transfection with up‐LNC000133 or vector lentivirus based on AGO2‐RIP; (H) Western blotting showing the protein expression of AGO2 in each group based on RNA pull‐down assay; (I) qRT‐PCR showing the mRNA expression of LNC000133 in each group based on RNA pull‐down assay. (A, B) *p < 0.05 compared with the control group; (D) ^#^ *p* < 0.05 compared with the WT + mimics group. (F) **p < 0.01 compared with the LNC000133 + IgG group, ^##^ *p* < 0.01 compared with the miR‐362‐5p + IgG group. (G) **p < 0.01 compared with the up‐LNC000133 + IgG group and vector + AGO2 group, ^##^ *p* < 0.01 compared with the vector + IgG group. (I) **p < 0.01 compared with NC‐probe group. AGO2, Argonaute 2; ceRNA, competing endogenous RNA; MUT, mutant‐type LNC000133; mimics, miR‐362‐5p mimics; inhibitor, miR‐362‐5p inhibitor; NC‐probe, negative control probe; RIP, RNA immunoprecipitation; WT, wild‐type LNC000133.

qRT‐PCR results indicated that upregulation of LNC000133 suppressed miR‐362‐5p expression, whereas downregulation of LNC000133 increased miR‐362‐5p expression. Conversely, transfection with miR‐362‐5p mimics reduced LNC000133 expression, while miR‐362‐5p inhibitors increased it, indicating a reciprocal negative regulatory relationship between LNC000133 and miR‐362‐5p (Figure 7A,B).

To verify their direct interaction, a dual‐luciferase reporter assay was performed. LNC000133 luciferase reporters were constructed by inserting sequences of wild‐type (WT) and mutant‐type (MUT, lacking the miR‐362‐5p binding sites) LNC000133 downstream of the luciferase gene (Figure 7C). Co‐transfection of miR‐362‐5p mimics with the WT LNC000133 reporter significantly reduced luciferase activity compared to the NC group (*p* < 0.01), indicating that miR‐362‐5p binds the predicted site on LNC000133. This inhibitory effect was absent in the MUT LNC000133 reporter due to the lack of the binding site. Furthermore, co‐transfection with miR‐362‐5p inhibitors reversed the suppressive effect of the mimics, confirming the specificity of this interaction (Figure 7D).

It was shown that the protein AGO2 could bind to lncRNAs and miRNAs and regulate the abundance of miRNAs in post‐transcription, thereby affecting the biological function of miRNAs [30, 31]. Therefore, to investigate whether AGO2 was involved in the modulation of miR‐362‐5p by LNC000133, we performed AGO2‐related RNA immunoprecipitation (AGO2‐RIP) to pull down endogenous miRNAs and lncRNAs bound to AGO2 and detected the expression of LNC000133 and miR‐362‐5p in the pull‐down complex by qRT‐PCR. Western blot analysis of AGO2‐RIP samples verified the successful immunoprecipitation of AGO2 (Figure 7E). qRT‐PCR results demonstrated that the AGO2‐RIP group showed markedly higher enrichment compared with the Input group, and in the LNC000133 overexpression group, AGO2 exhibited further enhanced enrichment of endogenous RNA (Figure 7F). In addition, endogenous LNC000133 and miR‐362‐5p were specifically enriched by the AGO2 protein in osteoblasts, but not by a control IgG protein (Figure 7G).

Furthermore, the RNA pull‐down assay demonstrated that both AGO2 protein and LNC000133 were specifically enriched by miR‐362‐5p probes, but not by NC‐probes (Figure 7H,I). Taken together, these findings revealed the interaction between LNC000133 and miR‐362‐5p in an AGO2‐dependent manner.

### 3.12 miR‐362‐5p Mediated the Activation of the TGF‐β3/SMAD3/BMP2 Signalling Pathway by LNC000133‐Modified hBMSCs‐Exos in the Proliferation and Osteogenic Differentiation of Osteoblasts

Our previous results demonstrated that LNC000133 promoted osteoblast proliferation and differentiation by activating the TGF‐β3/SMAD3/BMP2 signalling pathway, which was predicted as a downstream target of miR‐362‐5p in our bioinformatics analysis. Furthermore, dual‐luciferase assays confirmed the direct interaction between LNC000133 and miR‐362‐5p, suggesting that miR‐362‐5p serves as a molecular link between LNC000133 and the TGF‐β3/SMAD3/BMP2 pathway.

**FIGURE 8:**
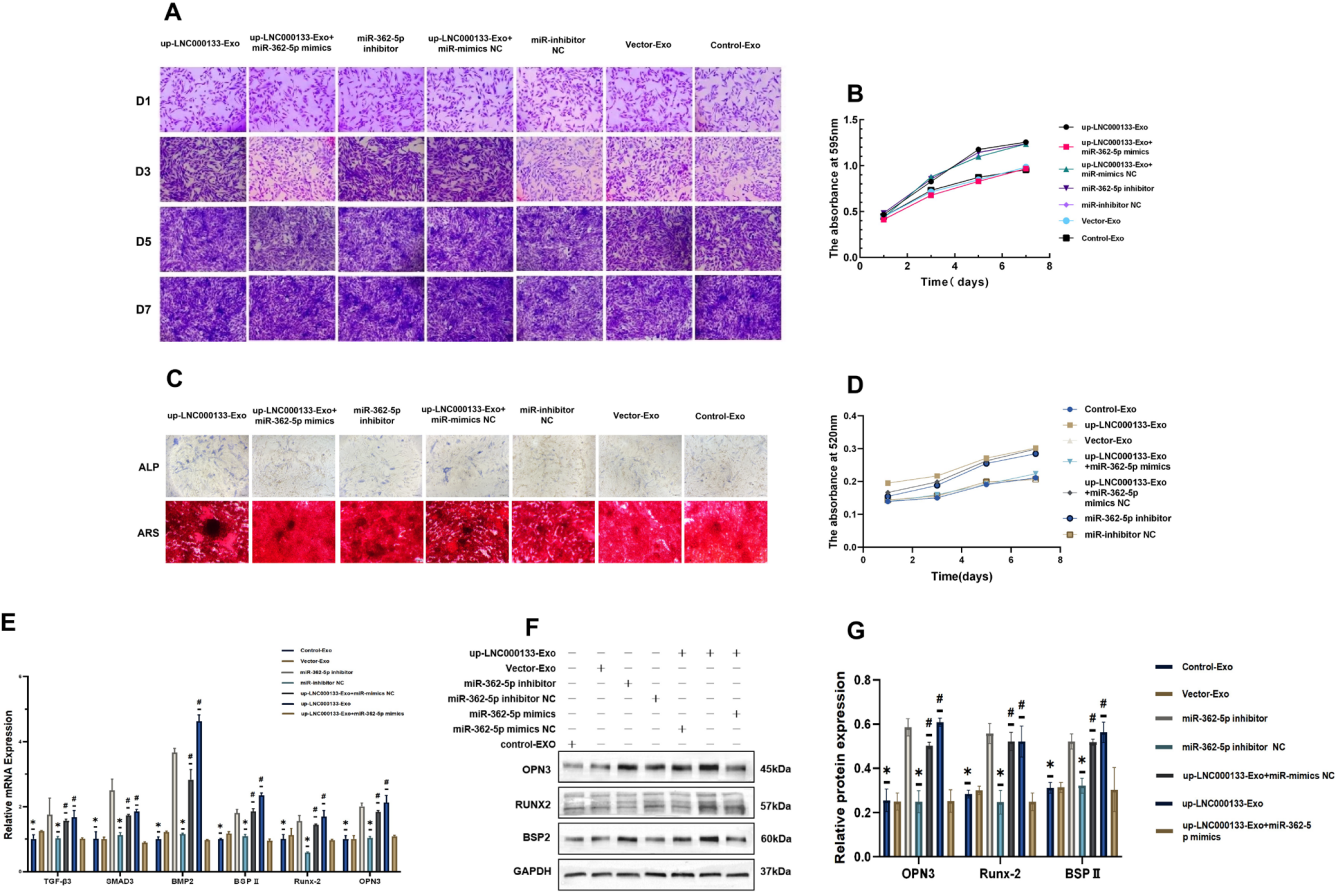
Results of miR‐362‐5p interference experiments. (A) Crystal violet staining; scale bar: 200 μm; (B) Proliferation viability of osteoblasts co‐incubated with hBMSCs‐Exos from day 1 to day 7, determined by crystal violet assay; (C) ARS staining and ALP staining of osteoblasts; scale bar: 100 μm; (D) ALP activity of osteoblasts from day 1 to day 7; (E) Expression levels of related mRNAs; (F) Representative Western blot bands of osteogenesis‐related proteins; (G) Quantification of osteogenesis‐related protein expression. *p < 0.05 compared with the miR‐362‐5p inhibitor group; ^#^ *p* < 0.05 compared with the up‐LNC000133‐Exo + miR‐362‐5p mimics group. ALP, alkaline phosphatase; ARS, Alizarin Red S.

Crystal violet staining revealed that both up‐LNC000133‐Exo and miR‐362‐5p inhibitor‐Exo significantly promoted osteoblast proliferation, whereas this effect was reversed by miR‐362‐5p mimics (Figure 8A,B). Similar trends were observed in Alizarin Red staining, ALP staining and activity assays (Figure 8C,D).

qRT‐PCR showed that LNC000133 overexpression downregulated miR‐362‐5p expression and upregulated the expression of TGF‐β3, SMAD3, BMP2 and osteogenic genes such as OPN3, Runx‐2 and BSP II, consistent with the effects of miR‐362‐5p inhibition. Notably, the effects of LNC000133 were blocked by miR‐362‐5p mimics (Figure 8E). Western blot analysis confirmed corresponding changes in osteogenic protein expression (Figure 8F,G).

## 4 Discussion

SONFH is the most prevalent form of non‐traumatic osteonecrosis of the femoral head, characterized by disrupted local blood supply caused by prolonged or high‐dose glucocorticoid use, leading to bone cell death and impaired tissue repair. The eventual collapse of the femoral head and joint dysfunction present the greatest challenge in treating SONFH, often necessitating THA. Therefore, early and effective bone repair is key to hip preservation treatment for this disease. However, definitive therapeutic targets for SONFH remain elusive, despite multiple proposed pathogenic mechanisms such as impaired blood supply, abnormal lipid metabolism, weakened osteogenic capacity of bone marrow mesenchymal stem cells (BMSCs), cell apoptosis and gene polymorphisms [32].

Our previous studies attempted to reveal the molecular targets for SONFH therapy, identifying many DEGs in hBMSCs related to SONFH and conducting preliminary validation [2, 11, 16, 22]. However, research on intercellular communication and information transmission remains limited. Exosomes, a type of extracellular vesicle and mediators of intercellular signalling, play an important role in stem cell therapy by carrying genetic material and proteins [33]. In this study, we obtained differential expression profiles of lncRNAs, miRNAs and mRNAs from hBMSCs‐Exos isolated from patients with SONFH and control patients with femoral neck fractures. Several novel lncRNAs associated with SONFH were identified through NGS. Further bioinformatics analysis showed that these DEGs were involved in cell proliferation, differentiation and exosome uptake, including processes such as cell cycle regulation, intracellular transport and post‐transcriptional gene regulation. Among the novel lincRNAs, LNC000133, a downregulated gene in hBMSCs from patients with SONFH, was selected for further validation because its co‐expressed and co‐located genes were involved in cell proliferation, osteogenic differentiation and endocytosis.

Firstly, the comprehensive biological characteristics of LNC000133 were identified using Northern blot, RACE, FISH and qRT‐PCR analysis. qRT‐PCR revealed significant downregulation of LNC000133 expression in both hBMSCs and hBMSCs‐Exos obtained from patients with SONFH compared to controls. In addition, Northern blot analysis confirmed the definite expression of LNC000133 in hBMSCs. These results validated the findings from NGS regarding LNC000133. Furthermore, the full‐length sequence of LNC000133 was obtained through RACE analysis, which facilitated downstream functional studies and vector construction, while also enriching current lncRNA databases. Moreover, FISH analysis showed that LNC000133 was localized in both the cytoplasm and nucleus in the majority of hBMSCs, suggesting it plays multifaceted roles in transcriptional regulation, mRNA stability and intracellular signalling [34, 35]. Together, these findings enhanced the annotation information of LNC000133 and provided important evidence for subsequent functional and mechanistic studies.

As two cell types coexisting in the microenvironment of the femoral head, the differentiation and mineralization capacities of osteoblasts and BMSCs are critical for bone regeneration and repair, especially in SONFH [36, 37]. Numerous studies have shown that bone repair and reconstruction in necrotic areas of SONFH can be promoted by regulating the functions of osteoblasts and BMSCs [2, 38, 39, 40]. Importantly, exosomes serve as a medium for information transmission and communication between BMSCs and osteoblasts. Specifically, osteoblasts, as target cells of BMSCs, can absorb hBMSCs‐Exos via endocytosis to exert biological functions such as osteogenesis, mineralization and cell proliferation [41, 42, 43, 44]. In this study, exposure to upregulated LNC000133‐modified hBMSCs‐Exos significantly increased exosome uptake by osteoblasts, which in turn enhanced their osteogenic differentiation and proliferation abilities. This was demonstrated by increased ALP activity, mineralized nodule formation and expression of key osteogenic markers including Runx‐2, BSP II and OPN3. Therefore, it is proposed that LNC000133 regulates the osteogenic differentiation and proliferation of osteoblasts via exosome‐mediated intercellular communication.

The proliferation and differentiation of osteoblasts is a complex BP regulated by numerous nucleic acids, proteins and signalling pathways [45, 46, 47, 48]. LncRNAs can exert their biological functions through various mechanisms [49, 50, 51, 52]. Among these, the ceRNA function of lncRNAs has attracted increasing attention in many studies [53, 54, 55]. Specifically, lncRNAs can act as molecular sponges to sequester specific miRNAs, thereby relieving their repression on downstream osteogenic genes. Notably, a targeted relationship between LNC000133 and miR‐362‐5p, as well as TGF‐β3, SMAD3 and BMP II, was predicted through bioinformatics analyses including structural matching, co‐expression and co‐localization. Based on the ceRNA theory, we constructed the LNC000133/miR‐362‐5p/TGF‐β3/SMAD3/BMP2 signalling pathway axis to further elucidate the molecular mechanism by which LNC000133‐modified hBMSCs‐Exos regulate osteoblast function.

Our study showed that TGF‐β3, SMAD3 and BMP2 were significantly upregulated during osteoblast proliferation and differentiation regulated by LNC000133‐modified hBMSCs‐Exos. However, this effect was significantly weakened by Pirfenidone, a specific inhibitor of the TGF‐β signalling pathway. This indicated that LNC000133‐modified hBMSCs‐Exos promoted the proliferation and osteogenesis of osteoblasts by activating the TGF‐β3/SMAD3/BMP2 signalling pathway. Subsequently, interference experiments targeting miR‐362‐5p were conducted in this study. We found that the regulatory effects of LNC000133‐modified hBMSCs‐Exos on osteoblasts were similar to those of the miR‐362‐5p inhibitor, while miR‐362‐5p mimics impaired the activation of the TGF‐β3/SMAD3/BMP2 pathway induced by LNC000133‐modified hBMSCs‐Exos. These results demonstrated that miR‐362‐5p mediated the LNC000133‐activated TGF‐β3/SMAD3/BMP2 signalling pathway to regulate the proliferation and osteogenesis of osteoblasts. Furthermore, we investigated the mutual regulation between LNC000133 and miR‐362‐5p and found a negative interaction between them in hBMSCs. Moreover, a dual‐luciferase reporter assay confirmed the targeted binding between LNC000133 and miR‐362‐5p, demonstrating that the specific sequence of miR‐362‐5p binds to LNC000133. In addition, previous studies have shown that lncRNAs can act as molecular sponges for miRNAs by competitively binding them within the AGO2‐mediated RNA‐induced silencing complex (RISC), thereby regulating their biological functions [30, 31]. In this context, we performed AGO2‐RIP and RNA pull‐down assays. The RIP results indicated that both LNC000133 and miR‐362‐5p were co‐enriched in AGO2‐associated complexes, suggesting that their interaction occurs within a canonical RISC‐mediated post‐transcriptional regulatory framework. In parallel, RNA pull‐down assays further supported the existence of a direct physical interaction between LNC000133 and miR‐362‐5p. Taken together, these findings suggest that LNC000133‐modified hBMSCs‐Exos promote the proliferation and osteogenesis of osteoblasts by specifically suppressing miR‐362‐5p, thereby activating the TGF‐β3/SMAD3/BMP2 signalling pathway.

In summary, this study identified differential expression profiles of lncRNAs, miRNAs and mRNAs in hBMSCs‐derived exosomes associated with SONFH and acquired and validated relevant biological information about the novel lncRNA LNC000133. More importantly, this study further elucidated the underlying mechanism by which LNC000133‐modified hBMSCs‐Exos promoted the proliferation and osteogenesis of osteoblasts by targeting the miR‐362‐5p/TGF‐β3/SMAD3/BMP2 signalling pathway axis.

## Author Contributions

**Chengbin Yang:** conceptualization, investigation, methodology, visualization and writing – original draft. **Tixiong Xia:** methodology, investigation, visualization, writing – original draft. **Xi Li:** investigation, methodology. **Tong Chen:** methodology, investigation. **Yingxing Xu:** conceptualization, investigation, methodology, funding acquisition, writing – review and editing, supervision.

## Funding

This work was supported by National Natural Science Foundation of China, 82260427, Science and Technology Plan Project of Yunnan Province Technology Hall, 202301AT070134, Yunnan Revitalization Talent Support Program, XDYC‐QNRC‐2023‐0198, Yunnan Province medical discipline reserve talent project, H‐2024030 and PhD Research Fund Project of the First Affiliated Hospital of Kunming Medical University, 2021BS016.

## Ethics Statement

The study was approved by ‘The Independent Ethics Committee’ of First Affiliated Hospital of Kunming Medical University (KMMU20220517). Informed Consent was taken from each of the patients for the treatment procedure.

## Consent

Consent for Publication was taken from the patients.

## Conflicts of Interest

The authors declare no conflicts of interest.

## Supporting information


**Figure S1:** Differential expression profiles of lncRNAs in hBMSCs‐Exos from SONFH patients and bioinformatics analysis. (A, B) Heatmap and volcano plot of differentially expressed lncRNAs (red: upregulated; blue: downregulated); (C, D) GO analysis of lncRNA co‐expression and co‐localization; (E, F) KEGG pathway analysis of lncRNA co‐expression and co‐localization; (G) Classification of lncRNAs; (H) Proportion of novel lncRNA. BP, Biological Process; CC, Cellular Component; MF, Molecular Function; GO, Gene Ontology; KEGG, Kyoto Encyclopedia of Genes and Genomes pathway analysis.

**Figure S2:** Expression profiles and functional analysis of miRNAs in SONFH. (A, B) Heatmap and volcano plot of differentially expressed miRNAs (red: upregulated; blue: downregulated); (C) GO analysis of miRNAs; (D) KEGG pathway analysis of miRNAs.

**Figure S3:** Expression profiles and functional analysis of mRNAs in SONFH. (A, B) Heatmap and volcano plot of differentially expressed mRNAs (red: upregulated; blue: downregulated); (C) GO analysis of mRNAs; (D) KEGG pathway analysis of mRNAs.

**Figure S4:** RACE showing the full‐length sequence of LNC000133.

**Figure S5:** Efficiency of transfection in hBMSCs for LNC000133 under a fluorescence microscope (scale bar = 100 μm).

**Table S1:** Amplification probe sequences for Northern blot analysis.

**Table S2:** Amplification probe sequences for Rapid Amplification of cDNA Ends (RACE) analysis.

**Table S3:** The sequences of primers for qRT‐PCR.

## Data Availability

The data that support the findings of this study are available from the corresponding author upon reasonable request.
