# Supplementary figures and images for: A Novel Long Noncoding RNA–LNC000133 Associated With Steroid‐Induced Osteonecrosis of the Femoral Head Promotes Osteoblast Differentiation Through Bone Marrow Mesenchymal Stem Cells‐Derived Exosomes Pathway: A Bioinformatics Validation and Detailed Mechanistic Study
Source: J Cell Mol Med. 2026 Apr 17;30(8):e71135. doi: 10.1111/jcmm.71135 (PMC13090172; doi:10.1111/jcmm.71135)

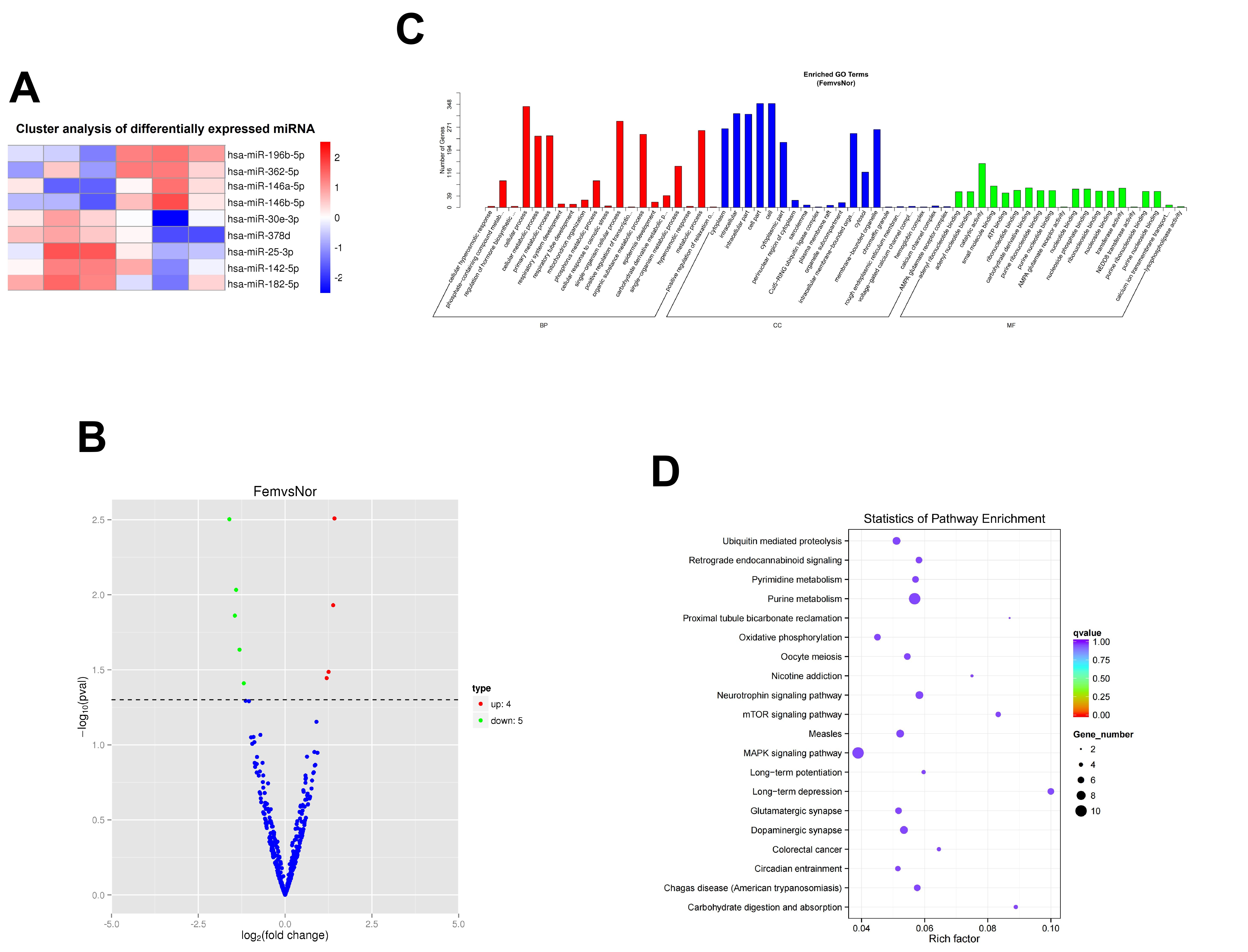

Supplement: Supplementary file 2 — Figure S2: Expression profiles and functional analysis of miRNAs in SONFH. (A, B) Heatmap and volcano plot of differentially expressed miRNAs (red: upregulated; blue: downregulated); (C) GO analysis of miRNAs; (D) KEGG pathway analysis of miRNAs. [file JCMM-30-e71135-s005.jpg]

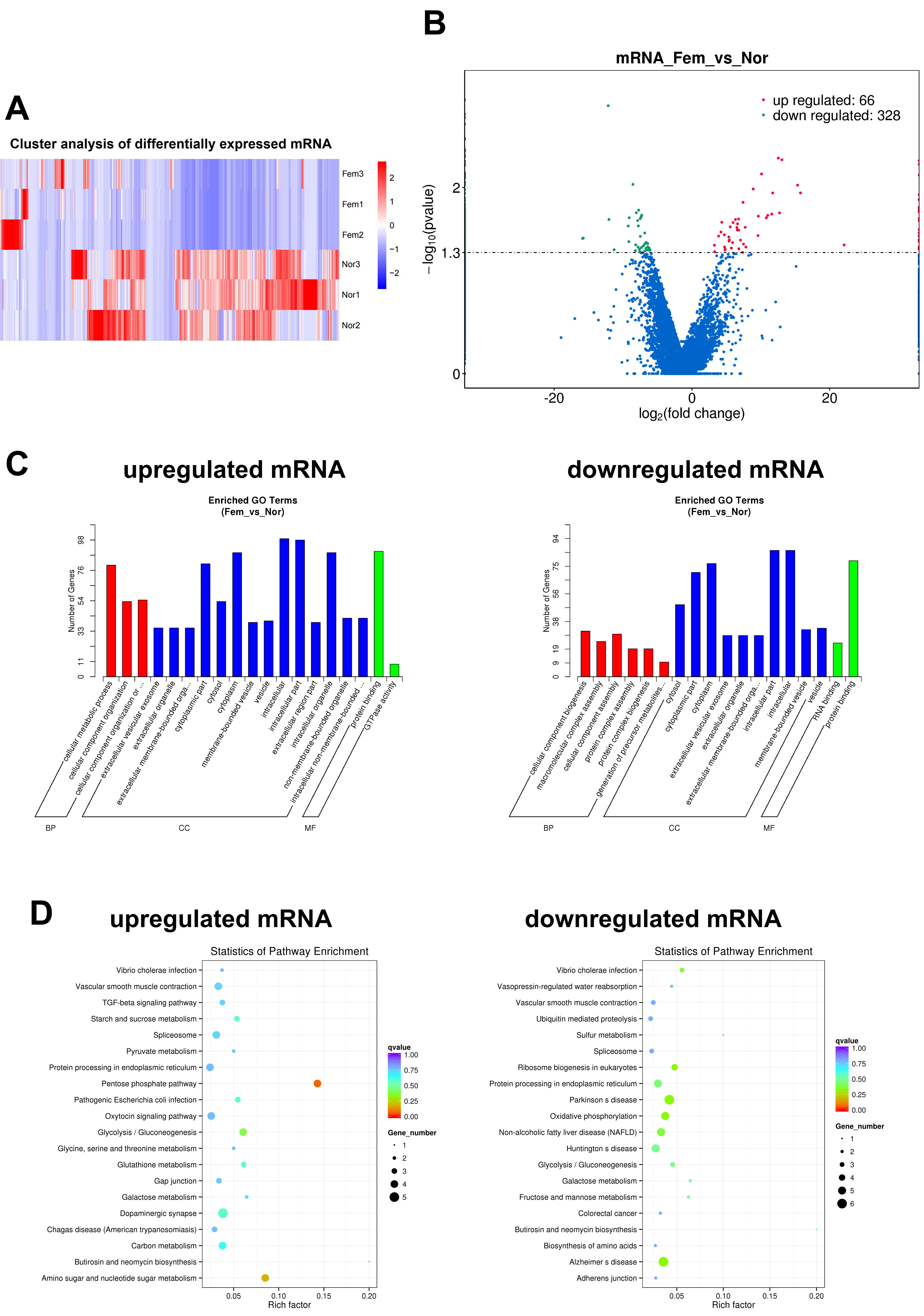

Supplement: Supplementary file 3 — Figure S3: Expression profiles and functional analysis of mRNAs in SONFH. (A, B) Heatmap and volcano plot of differentially expressed mRNAs (red: upregulated; blue: downregulated); (C) GO analysis of mRNAs; (D) KEGG pathway analysis of mRNAs. [file JCMM-30-e71135-s002.jpg]

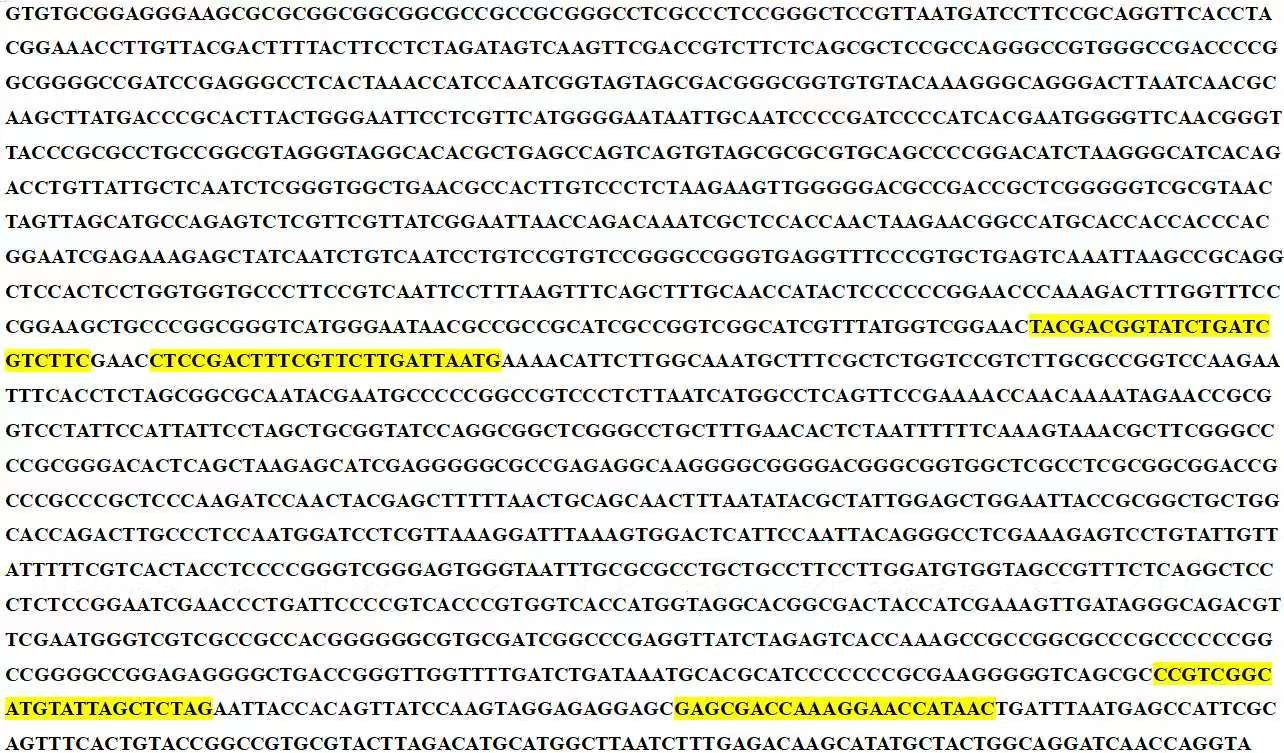

Supplement: Supplementary file 4 — Figure S4: RACE showing the full‐length sequence of LNC000133. [file JCMM-30-e71135-s008.jpg]

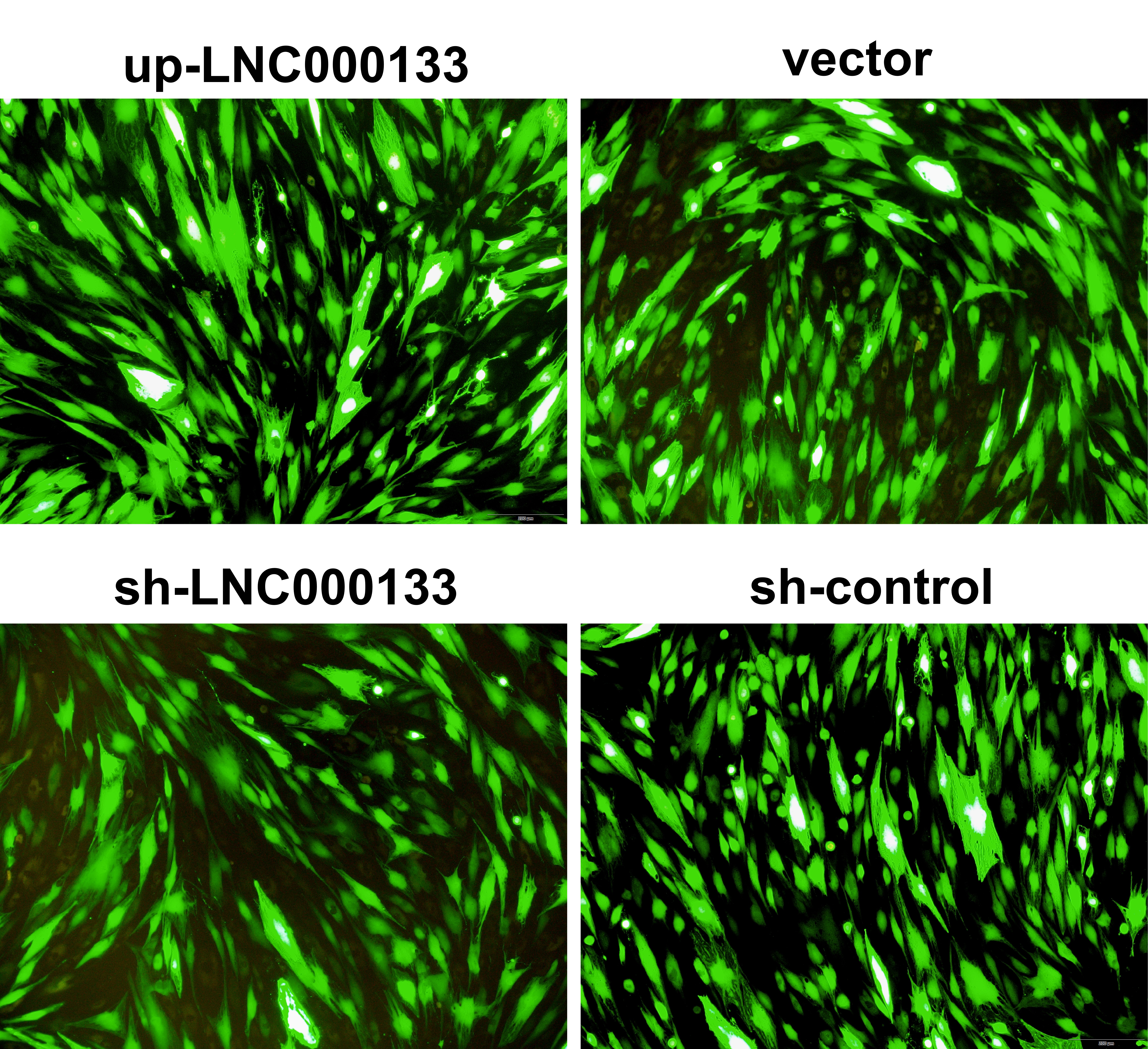

Supplement: Supplementary file 5 — Figure S5: Efficiency of transfection in hBMSCs for LNC000133 under a fluorescence microscope (scale bar = 100 μm). [file JCMM-30-e71135-s001.jpg]
