# Supplementary material for: A Novel Long Noncoding RNA–LNC000133 Associated With Steroid‐Induced Osteonecrosis of the Femoral Head Promotes Osteoblast Differentiation Through Bone Marrow Mesenchymal Stem Cells‐Derived Exosomes Pathway: A Bioinformatics Validation and Detailed Mechanistic Study
Source: J Cell Mol Med. 2026 Apr 17;30(8):e71135. doi: 10.1111/jcmm.71135 (PMC13090172; doi:10.1111/jcmm.71135)
Supplement: Supplementary file 6 — Table S1: Amplification probe sequences for Northern blot analysis. [file JCMM-30-e71135-s004.docx]

**Supplementary Table S1: Amplification probe sequences for Northern blot analysis**

| **Gene** | **Primer sequence (5'-3')** |
| --- | --- |
| SNB517 | CGGCATCGTTTATGGTCG |
| SNB517 | TGGAGGGCAAGTCTGGTG |
