# Supplementary material for: A Novel Long Noncoding RNA–LNC000133 Associated With Steroid‐Induced Osteonecrosis of the Femoral Head Promotes Osteoblast Differentiation Through Bone Marrow Mesenchymal Stem Cells‐Derived Exosomes Pathway: A Bioinformatics Validation and Detailed Mechanistic Study
Source: J Cell Mol Med. 2026 Apr 17;30(8):e71135. doi: 10.1111/jcmm.71135 (PMC13090172; doi:10.1111/jcmm.71135)
Supplement: Supplementary file 7 — Table S2: Amplification probe sequences for Rapid Amplification of cDNA Ends (RACE) analysis. [file JCMM-30-e71135-s007.docx]

**Supplementary Table S2: Amplification probe sequences for Rapid Amplification of cDNA Ends (RACE) analysis**

1. **Adaptor Primers**

| **Type** | **Primer sequence**（5′→3′） |
| --- | --- |
| 5’ adaptor | GCTGTCAACGATACGCTACGTAACGGCATGACAGTGGGIIGGGIIGGGIIG |
| 3’ adaptor | GCTGTCAACGATACGCTACGTAACGGCATGACAGTGTTTTTTTTTTTTTTTTTT |
| 5.3’ outer | GCTGTCAACGATACGCTACGTAAC |
| 5.3’ inner | GCTACGTAACGGCATGACAGTG |

1. **Internal Amplification Primers**

| **Primer Name** | **Primer sequence（5′→3′）** |
| --- | --- |
| RC1078-Y1-F | TTAAGTTTCAGCTTTGCAACCATAC |
| RC1078-Y1-R | CCCTGTAATTGGAATGAGTCCACT |
| RC1078-Y2-F | GATCCAACTACGAGCTTTTTAACTG |
| RC1078-Y2-R | CTTACCTACCTGGTTGATCCTGC |

1. **Specific Primers for 3′RACE**

| **Primer Name** | **Primer sequence（5′→3′）** |
| --- | --- |
| RC1078-FT1 | AGGCACGGCGACTACCATC |
| RC1078-FT2 | CTGCTGCCTTCCTTGGATGT |
| RC1078-F1 | CCGTCGGCATGTATTAGCTCTAG |
| RC1078-F2 | GAGCGACCAAAGGAACCATAAC |

1. **5′RACE RT and Specific Primers**

| **Primer Name** | **Primer sequence（5′→3′）** |
| --- | --- |
| RC1078-RT1 | TAGCGTATATTAAAGTTGCTGCAGT |
| RC1078-RT2 | GAAGCGTTTACTTTGAAAAAATTAGAG |
| RC1078-R2 | CATTAATCAAGAACGAAAGTCGGAG |
| RC1078-R3 | GAAGACGATCAGATACCGTCGTA |
