# Supplementary material for: A Novel Long Noncoding RNA–LNC000133 Associated With Steroid‐Induced Osteonecrosis of the Femoral Head Promotes Osteoblast Differentiation Through Bone Marrow Mesenchymal Stem Cells‐Derived Exosomes Pathway: A Bioinformatics Validation and Detailed Mechanistic Study
Source: J Cell Mol Med. 2026 Apr 17;30(8):e71135. doi: 10.1111/jcmm.71135 (PMC13090172; doi:10.1111/jcmm.71135)
Supplement: Supplementary file 8 — Table S3: The sequences of primers for qRT‐PCR. [file JCMM-30-e71135-s003.docx]

**Supplementary Table S3 The sequences of primers for qRT-PCR**

| Gene | Primer sequence (5'-3') |
| --- | --- |
| LNC000133 | F：CTTTCGCTCTGGTCCGTCTTG |
|  | R：CTCGATGCTCTTAGCTGAGTGTCC |
| mi-362-5p | AAUCCUUGGAACCUAGGUGUGAGU |
| TGF-β3 | F：ACTTGCACCACCTTGGACTTC |
|  | R：GGTCATCACCGTTGGCTCA |
| SMAD3 | F：TGGACGCAGGTTCTCCAAAC |
|  | R：CCGGCTCGCAGTAGGTAAC |
| BMP2 | F：GACATCCTGAGCGAGTTCGA |
|  | R：CACTTGTTTCTGGCAGTTCTTC |
| BSP Ⅱ | F：ACAATCCGTGCCACTCACT |
|  | R：TTTCATCGAGAAAGCACAGG |
| OPN3 | F：CGAAGATCCCTTTTGCAGCTT |
|  | R：ATGGGTCTGATCTGCATTTCACT |
| Runx-2 | F：CCGCCTCAGTGATTTAGGGC |
|  | R：GGGTCTGTAATCTGACTCTGTCC |
| GAPDH | F：GCTGGGAGGTGTTCGACATC |
|  | R：CACGGTCTTATCGTCCTGGC |
| U6 | F：CTCGCTTCGGCAGCACATATACTA |
|  | R：ACGAATTTGCGTGTCATCCTTGCG |

qRT-PCR quantitative real-time PCR, TGF-β3 Transforming Growth Factor Beta 3, SMAD3 SMAD Family Member 3, BMP2 Bone Morphogenetic Protein 2, BSPII bone sialoprotein II, OPN3 Opsin 3, Runx-2 runtrelated transcription factor-2.
